# MicroRNA miR-214 Inhibits Snakehead Vesiculovirus Replication by Promoting IFN-α Expression *via* Targeting Host Adenosine 5′-Monophosphate-Activated Protein Kinase

**DOI:** 10.3389/fimmu.2017.01775

**Published:** 2017-12-11

**Authors:** Chi Zhang, Shuangshuang Feng, Wenting Zhang, Nan Chen, Abeer M. Hegazy, Wenjie Chen, Xueqin Liu, Lijuan Zhao, Jun Li, Li Lin, Jiagang Tu

**Affiliations:** ^1^Department of Aquatic Animal Medicine, College of Fisheries, Huazhong Agricultural University, Wuhan, China; ^2^Guangzhou Key Laboratory of Aquatic Animal Diseases and Waterfowl Breeding, Guangdong Provincial Key Laboratory of Waterfowl Healthy Breeding, College of Animal Sciences and Technology, Zhongkai University of Agriculture and Engineering, Guangzhou, China; ^3^Key Laboratory of Prevention and Control Agents for Animal Bacteriosis, Institute of Animal Husbandry and Veterinary, Hubei Academy of Agricultural Sciences, Wuhan, China; ^4^Central Laboratory for Environmental Quality Monitoring (CLEQM), National Water Research Center (NWRC), Cairo, Egypt; ^5^School of Biological Sciences, Lake Superior State University, Sault Ste. Marie, MI, United States; ^6^Laboratory for Marine Fisheries Science and Food Production Processes, Qingdao National Laboratory for Marine Science and Technology, Qingdao, China; ^7^Hubei Engineering Technology Research Center for Aquatic Animal Diseases Control and Prevention, Huazhong Agricultural University, Wuhan, China

**Keywords:** snakehead vesiculovirus, microRNA, miR-214, interferon, replication, adenosine 5′-monophosphate-activated protein kinase

## Abstract

**Background:**

Snakehead vesiculovirus (SHVV), a new rhabdovirus isolated from diseased hybrid snakehead, has emerged as an important pathogen during the past few years in China with great economical losses in snakehead fish cultures. However, little is known about the mechanism of its pathogenicity. MicroRNAs are small noncoding RNAs that posttranscriptionally modulate gene expression and have been indicated to regulate almost all cellular processes. Our previous study has revealed that miR-214 was downregulated upon SHVV infection.

**Results:**

The overexpression of miR-214 in striped snakehead (SSN-1) cells inhibited SHVV replication and promoted IFN-α expression, while miR-214 inhibitor facilitated SHVV replication and reduced IFN-α expression. These findings suggested that miR-214 negatively regulated SHVV replication probably through positively regulating IFN-α expression. Further investigation revealed that adenosine 5′-monophosphate-activated protein kinase (AMPK) was a target gene of miR-214. Knockdown of AMPK by siRNA inhibited SHVV replication and promoted IFN-α expression, suggesting that cellular AMPK positively regulated SHVV replication and negatively regulated IFN-α expression. Moreover, we found that siAMPK-mediated inhibition of SHVV replication could be partially restored by miR-214 inhibitor, indicating that miR-214 inhibited SHVV replication at least partially *via* targeting AMPK.

**Conclusion:**

The findings of this study complemented our early study, and provide insights for the mechanism of SHVV pathogenicity. SHVV infection downregulated miR-214, and in turn, the downregulated miR-214 increased the expression of its target gene AMPK, which promoted SHVV replication via reducing IFN-α expression. It can therefore assume that cellular circumstance with low level of miR-214 is beneficial for SHVV replication and that SHVV evades host antiviral innate immunity through decreasing IFN-α expression *via* regulating cellular miR-214 expression.

## Introduction

MicroRNAs (miRNAs) are a class of small (~22 nt) noncoding RNAs that posttranscriptionally degrade and/or suppress translation of target mRNAs through base pairing between the “seed sequences” (2–8 nt at the 5′ end) of miRNAs and the target transcripts (1–5). Host miRNAs, typically binding to the 3′ untranslated regions (UTRs) of target transcripts (5–10), have been reported to play important roles in the regulation of virus replication (1, 5, 7, 10, 11). Moreover, the regulatory roles of miRNAs in virus replication were even utilized by viruses to promote their replication (6, 9). Therefore, understanding the roles of miRNAs in virus infection is helpful for understanding the mechanisms of virus pathogenesis.

Snakehead vesiculovirus (SHVV) is a fish rhabdovirus isolated from diseased hybrid snakehead in 2014 in China (12). It has caused high mortality to cultured snakehead fish these yeas. Up to now, the study about the mechanism of its pathogenicity is limited. SHVV belongs to the genus *Perhabdovirus*, family *Rhabdoviridae* (13). Its genome is an ~11 kb negative-sense RNA molecule that encodes five proteins: nucleoprotein (N), phosphoprotein (P), matrix protein (M), glycoprotein (G), and RNA-dependent RNA polymerase protein (L) (12). Our previous study has revealed that SHVV infection downregulated miR-214 (14), and in turn, miR-214 could inhibit SHVV production by targeting viral N and P (15). However, it is unclear whether miR-214 can regulate SHVV replication *via* targeting host factors that are required for SHVV replication. MiR-214 has recently been observed to be upregulated by Vibro harveyi, and the upregulated miR-214 inhibited the production of inflammatory cytokines by targeting host myd88 (16). Consequently, miR-214 played important roles in regulating pathogens infection.

Adenosine 5′-monophosphate-activated protein kinase (AMPK) is a heterotrimeric serine/threonine kinase (17), which is considered as pivotal regulator of host cellular metabolism *via* sensing cellular energy status (18). When the energy levels in cells decrease, AMPK is activated through phosphorylation by an upstream kinase (18). Activated AMPK thereby downregulates anabolic processes that consume ATP and upregulates catabolic processes that synthesize ATP (18). Given the role of sensing changes of cellular energy status, it is not surprising that AMPK plays an important role in virus infection (19). However, growing evidences have revealed that viruses can modulate the activity of AMPK, and in turn, AMPK affects virus infection by regulating cellular autophagy or innate immunity (20, 21). Here, we reported that AMPK was a target gene of miR-214, negative regulator of IFN-α expression, and positive regulator of SHVV replication. Moreover, we determined that miR-214 could inhibit SHVV replication by promoting IFN-α expression *via* reducing AMPK expression. This study provided information for understanding the molecular mechanism of SHVV pathogenicity and a potential antiviral strategy against SHVV infection.

## Materials and Methods

### Cells and Viruses

Striped snakehead (SSN)-1 cells were maintained at 25°C in minimum essential medium (MEM) (HyClone, USA) supplemented with 10% heat-inactivated fetal bovine serum (FBS) (Gibco, New Zealand), penicillin (100 µg/ml), and streptomycin (100 µg/ml). SHVV was isolated from diseased hybrid snakehead fish and stored at −80°C.

### Reagents and Antibodies

The miR-214 mimic, miR-214 inhibitor, negative control (NC) mimic, and NC inhibitor were purchased from GenePharma (Shanghai, China). Their sequences were previously described (15). Two siRNAs for AMPK (accession number: MF989224) were synthesized from GenePharma (Shanghai, China). The sequences of the first one were: 5′-CCUCCAGUAUCAAGAUCUUTT-3′ (forward) and 5′-AAGAUCUUGAUACUGGAGGTT-3′ (reverse); the sequences of the second one were: 5′-GGACACGCCCAUUAUUAAATT-3′ (forward) and 5′-UUUAAUAAUGGGCGUGUCCTT-3′ (reverse).

The antibodies against G protein of SHVV and AMPK were produced and stored in our laboratory. The antibody against β-actin was purchased from Bioss Biotechnology Co., LTD. (Beijing, China). The secondary antibody donkey anti-rabbit IgG antibody was purchased from Gene Co., LTD. (Shanghai, China).

### Plasmids

The luciferase reporter plasmid pmirGLO-AMPK was constructed by amplifying the miR-214 target sequence (~200 nt) in the 3′ UTR of AMPK and cloning into vector pmirGLO with primers listed in Table 1. The plasmids pmirGLO-AMPK-MUT1 and pmirGLO-AMPK-MUT2 were generated by PCR mediated mutations into plasmid pmirGLO-AMPK using primers listed in Table 1. The expression plasmid p3XFLAG-CMV-14-AMPK was constructed by amplifying the open reading frame of AMPK gene and cloning into vector p3XFLAG-CMV-14 using primers listed in Table 1.

**Table 1:** Primers used in this study.

Application	Primer	Sequence (5′-3′)
qRT-PCR	SHVV-G-FW	ACACCATACATGCCAGAGGC
	SHVV-G-BW	GCCTCGCTGGGTATCCAAAT
	AMPK-FW	GCAGGAAGGAGGATAGAA
	AMPK-BW	GCAACTGAGCCCGTAAAA
	IFN-α-FW	TGTACCTCGGCCTTCTCGAT
	IFN-α-BW	CGAAGCCTGCAACTGGATGA
	β-actin-FW	CACTGTGCCCATCTACGAG
	β-actin-BW	CCATCTCCTGCTCGAAGTC
	miR-214-F	CGGACAGCAGGCACAGACAGGCAAAAA
	5SrRNA-F	GGAGACCGCCTGGGAATA

Reporter plasmids	AMPK-FW	CTAGCTAGC GTTGCTGCTCCGTTTC
	AMPK-BW	GCTCTAGA CGAGTCCTTCTCACCC
	AMPK-MUT1-FW	AGTCGTCTAAGGCGGAAAGGACGACAAATTTAAGGCAGAAA
	AMPK-MUT1-BW	TC GTCGTCCTTTCCGCCTTAGACGACG
	AMPK-MUT2-FW	TTAAATTTTTTTATAGATGTTTCAAACATCGGACGACAAAAA
	AMPK-MUT2-BW	AACTAAATTATAGTCGGTCGTCCGATGTTGAAACCTTTTAA

Expression plasmid	AMPK-FW-1	GGGGTACCAATGGGCAGCACGGCGGC
	AMPK-BW-1	GCTCTAGA CGTGCTCTCTCTGCCTTCTTTT

### Transfection

The mimics, inhibitors, or plasmids were incubated with TransIntroTM EL Transfection Reagent (TransGen Biotech, China) in 500 μl Opti-MEM medium (Invitrogen, USA) for 30 min at room temperature. The incubated samples were then put onto the SSN-1 cells. At 6 h post of transfection, the medium was replaced by 1 ml of MEM and continued incubation at 25°C.

### Dual-Luciferase Reporter Assay

The dual-luciferase reporter assay was performed as described previously (15). In brief, SSN-1 cells were co-transfected with NC mimic, miR-214 mimic, NC inhibitor, or miR-214 inhibitor, together with the luciferase reporter plasmids using TransIntroTM EL Transfection Reagent (TransGen Biotech, China). At 24 h post of transfection, the *Renilla* and firefly luciferase activities were measured, and the data were expressed as relative firefly luciferase activity normalized to *Renilla* luciferase activity.

### Virus Infection and Titration

Virus infection and titration experiments were performed as previously described (15). In brief, SSN-1 cells were incubated with SHVV for 2 h, the inoculum was then removed and the cells were washed twice with PBS followed by adding MEM medium with 5% FBS. At 24 h post of infection (poi), the supernatants were collected for virus titration by 50% tissue culture infectious dose (TCID_50_), and the cells were harvested for the detection of viral mRNA or host miR-214 by qRT-PCR with primers listed in Table 1.

### Quantitative RT-PCR of Viral mRNA, Host IFN-α mRNA, and miR-214

Total RNAs were extracted from cells with TRIzol reagent (Invitrogen) according to manufacturer’s instructions. The detection of viral mRNA, host IFN-α (accession number: MF989225) mRNA, and miR-214 was performed by qRT-PCR as previously described (15). Two sets of data were normalized using the 2^−ΔΔCt^ method. For the detection of viral mRNA, data was normalized to the level of β-actin in each sample, while for the miR-214 detection, the expression level of miR-214 was calculated after normalization to 5S rRNA.

### Western Blotting

Western blotting was performed as previously described (15). In brief, the extracted proteins were transferred onto a nitrocellulose membrane (Biosharp, China), which were blocked with 5% skim milk in tris-buffered saline with tween 20 (TBST) at 4°C overnight, followed by incubation with the primary antibody of SHVV protein (1:1,000) or β-actin (1:1,000) for 2 h at room temperature. The membranes were then washed three times with TBST and then incubated with IRDye 800CW conjugated donkey anti-rabbit antibody (1:10,000) for 1 h at room temperature. The signal intensity was then determined using Odyssey CLx (LI-COR, USA).

### Statistical Analysis

All statistical analyses were performed using Graphpad Prism 5.0 (GraphPad Software, CA, USA). The statistical significance of the data was determined by Student’s *t* test, and *P* < 0.05 was considered statistically significant. For data sets in which multiple comparisons were being made, the Student’s *t*-test was corrected by using false discovery rate.

## Results

### The Effect of miR-214 on the Transcription, Translation, and Production of SHVV

**Figure 1:**
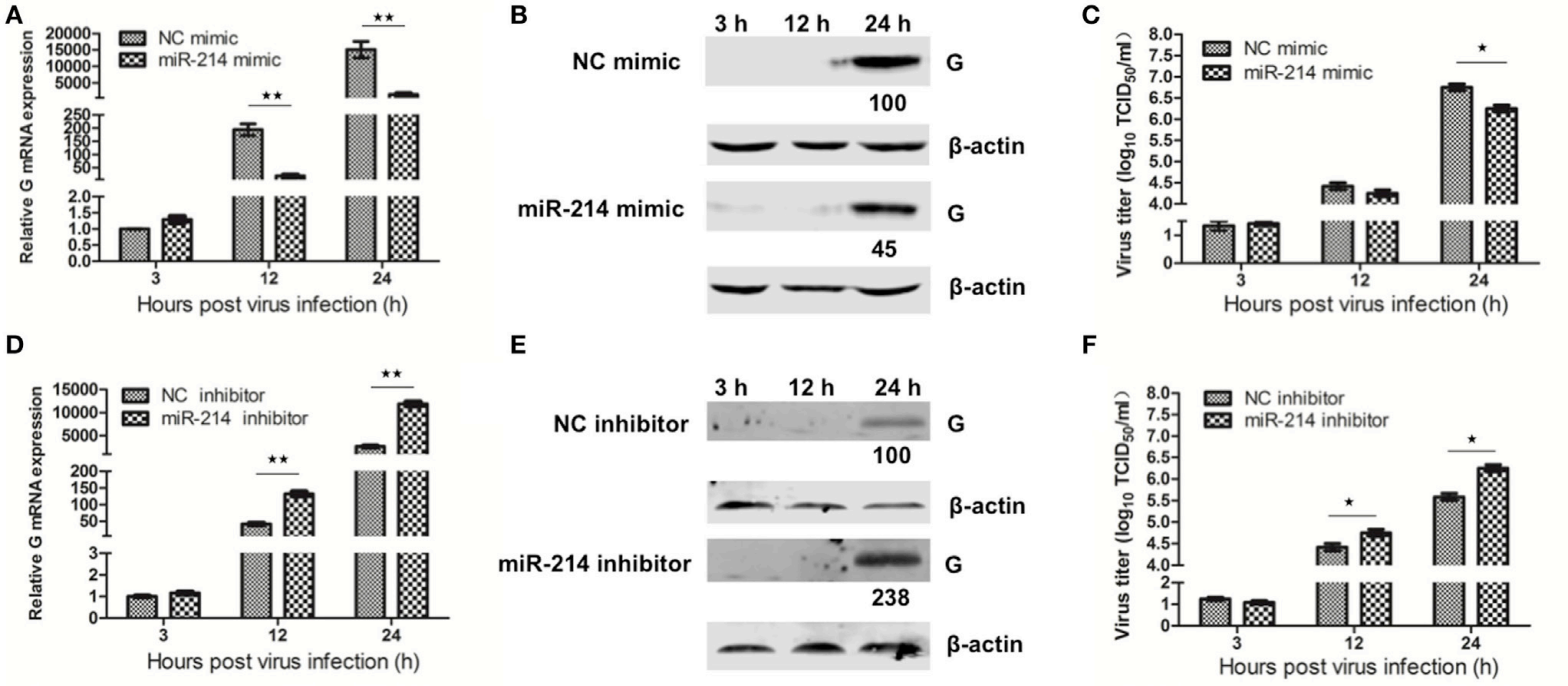
The effect of miR-214 on the transcription, translation, and production of snakehead vesiculovirus (SHVV). Striped snakehead (SSN)-1 cells were transfected with negative control (NC) mimic, miR-214 mimic, NC inhibitor, or miR-214 inhibitor, followed by SHVV infection. The cells and supernatants were harvested at various time points. **(A,D)** G mRNA in SSN-1 cells was measured using qRT-PCR. β-actin was used as the internal control. **(B,E)** G Protein was determined by western blot. β-actin was used as the internal control. The integrated optical densities of the protein bands were measured using Image-Pro Plus 6.0. The values of the G protein bands were normalized to that of β-actin. The values of the G protein bands in cells transfected with NC mimic or inhibitor were set as 100, respectively. **(C,F)** The SHVV titers in the supernatants were measured using TCID_50_. All the data are representative of at least two independent experiments, with each determination performed in triplicate (mean ± SD). The * and **, respectively, indicate statistically significant differences (*P < 0.05; **P < 0.01).

MiR-214 has been indicated to inhibit the replication of several human and mammalian viruses, including human cytomegalovirus (HCMV), murine cytomegalovirus (MCMV), and herpes simplex virus 1 (HSV-1) (22). In the same vein, our previous study has suggested that miR-214 inhibited SHVV replication (15). In this study, we further evaluated the effect of miR-214 on the transcription, translation, and production of SHVV at different time point poi. SSN-1 cells were transfected with miR-214 mimic, NC mimic, miR-214 inhibitor, or NC inhibitor, followed by SHVV infection. At 3, 12, and 24 h poi, the cells and supernatants were collected in order to detect viral G mRNA, G protein, and viral titer by qRT-PCR, western blot, and TCID_50_, respectively. As shown in Figure 1, viral G mRNA expression was not significantly altered at 3 h poi. However, at 12 and 24 h poi, overexpression of miR-214 significantly reduced, whereas miR-214 inhibitor increased, G mRNA level (Figures 1A,D). The expression of viral G protein was under detection at 3 and 12 h poi. At 24 h poi, it was apparent from Figures 1B,E that G protein expression was decreased by about 50% or increased to about 2.5-fold when the cells were transfected with miR-214 mimic or miR-214 inhibitor, respectively. Similar to G mRNA and G protein, the viral titers were reduced by transfection of miR-214 mimic and increased by transfection of miR-214 inhibitor (Figures 1C,F). Taken together, these findings demonstrate that miR-214 inhibits SHVV replication.

### miR-214 Promotes IFN-α Expression

**Figure 2:**
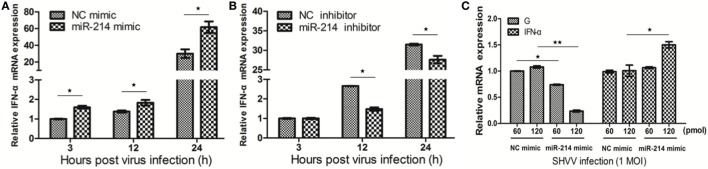
MiR-214 promotes IFN-α production. **(A,B)** Striped snakehead (SSN)-1 cells were transfected with negative control (NC) mimic, miR-214 mimic, NC inhibitor, or miR-214 inhibitor, followed by snakehead vesiculovirus (SHVV) infection. The cells were harvested at different time point. The IFN-α mRNA in SSN-1 cells was measured using qRT-PCR. β-actin was used as the internal control. **(C)** SSN-1 cells were transfected with different doses of NC mimic or miR-214 mimic, followed by SHVV infection. At 24 h poi, the cells were harvested. The G mRNA and IFN-α mRNA in SSN-1 cells were measured using qRT-PCR. β-actin was used as the internal control. All the data are representative of at least two independent experiments, with each determination performed in triplicate (mean ± SD). The * and **, respectively, indicate statistically significant differences (*P < 0.05; **P < 0.01).

Our previous study has revealed that miR-214 promoted IFN-α expression during SHVV infection by targeting SHVV P protein, an IFN-α antagonist (15). In this study, we further investigated the role of miR-214 in IFN-α expression at different time point post of SHVV infection. SSN-1 cells were transfected with miR-214 mimic, NC mimic, miR-214 inhibitor, or NC inhibitor, followed by SHVV infection. At 3, 12, and 24 h poi, IFN-α mRNA was detected using qRT-PCR. As shown in Figures 2A,B, overexpression of miR-214 significantly increased, whereas miR-214 inhibitor reduced, IFN-α mRNA at 12 and 24 h poi. Moreover, we found that overexpression of miR-214 inhibited SHVV replication and increased IFN-α mRNA in a dose-dependent manner (Figure 2C). In addition, we found that overexpression of miR-214 promoted poly (I:C)-induced IFN-α mRNA (Figure S1 in Supplementary Material). Our data suggest that the promotion of IFN-α expression by miR-214 could be the cause of its inhibition of SHVV replication.

### miR-214 Targets the 3′ UTR of AMPK mRNA

**Figure 3:**
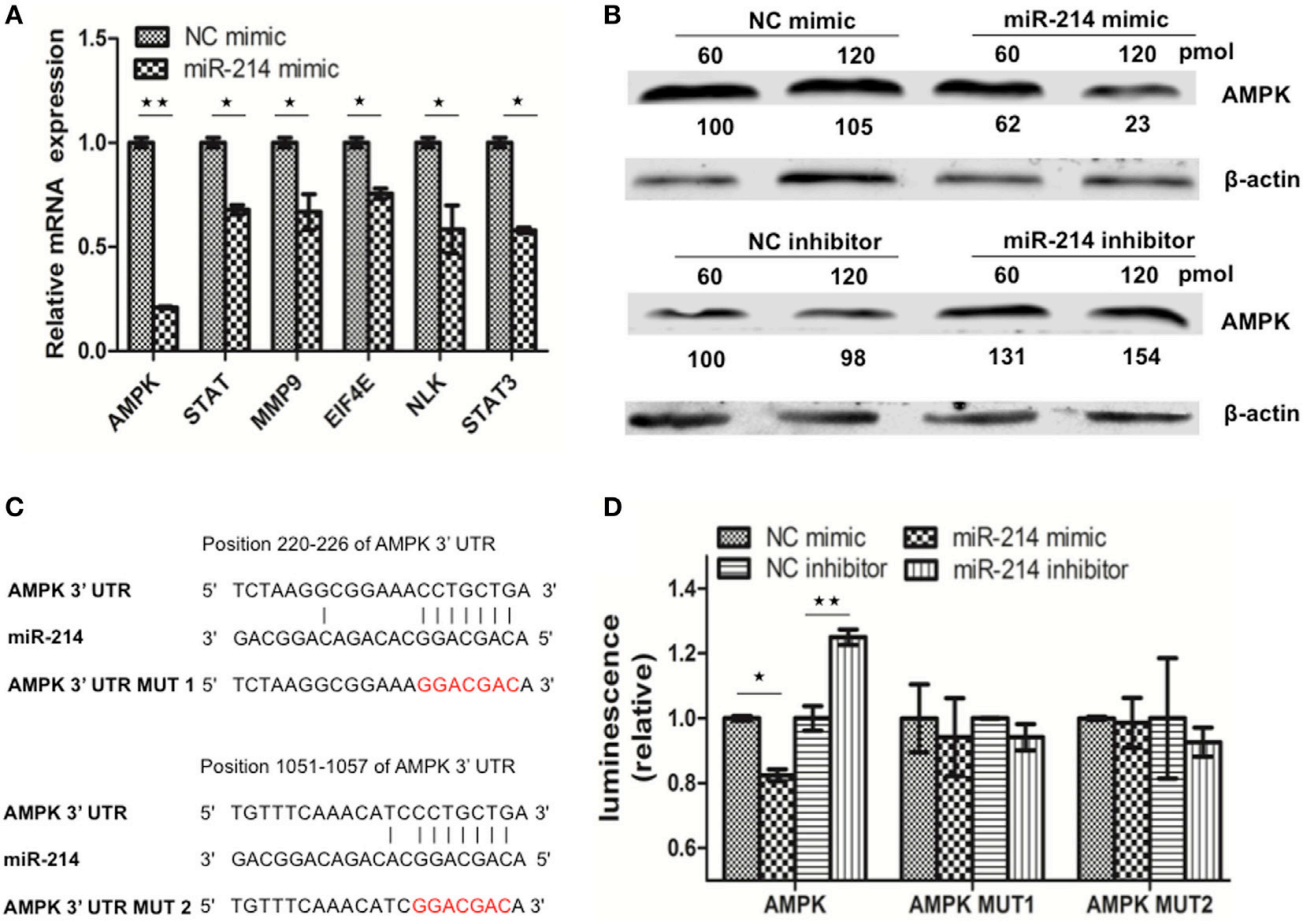
MiR-214 targets to the 3′ UTR of adenosine 5′-monophosphate-activated protein kinase (AMPK) mRNA. **(A)** qRT-PCR was used to evaluate the expression of six candidate target genes of miR-214. **(B)** Striped snakehead (SSN)-1 cells were transfected with different doses of negative control (NC) mimic, miR-214 mimic, NC inhibitor, or miR-214 inhibitor. At 24 h post of transfection, AMPK protein was determined by western blot. β-actin was used as the internal control. The integrated optical densities of the protein bands were measured using Image-Pro Plus 6.0. The values of the AMPK protein bands were normalized to that of β-actin. The values of the AMPK protein bands in cells transfected with 60 pmol of NC mimic or inhibitor were set as 100, respectively **(C)** Alignment of miR-214 with the predicted target sequences in the 3′ UTR of AMPK mRNA. **(D)** SSN-1 cells were transfected with pmirGLO-AMPK, pmirGLO-AMPK-MUT1, or pmirGLO-AMPK-MUT2, together with NC mimic, miR-214 mimic, NC inhibitor, or miR-214 inhibitor. Luciferase activity was measured at 24 h post of transfection, and the data were expressed as relative firefly luciferase activity normalized to Renilla luciferase activity. All the data are representative of at least two independent experiments, with each determination performed in triplicate (mean ± SD). The * and **, respectively, indicate statistically significant differences (*P < 0.05; **P < 0.01).

In addition to targeting viral P gene, it’s speculated that miR-214-mediated inhibition of SHVV replication and promotion of IFN-α expression could also be caused by targeting host genes. To determine host target genes of miR-214, high throughput transcriptomic sequencing of SSN-1 cells transfected with miR-214 mimic or NC mimic has been performed. The results showed that overexpression of miR-214 resulted in 1,301 upregulated genes and 1,613 downregulated genes (data not shown). Based on association possibility with virus replication, six downregulated genes were selected for further validation using qRT-PCR as follows (Table 2): signal transducers and activators of transcription (STAT), matrix metallopeptidase 9 (MMP9), eukaryotic translation initiation factor 4E (EIF4E), nemo Like Kinase (NLK), signal transducer and activator of transcription 3 (STAT3), and AMPK. Among them, AMPK was the most downregulated gene (Figure 3A). To further determine the effect of miR-214 on AMPK expression, SSN-1 cells were transfected with 60 and 120 pmol of miR-214 mimic, NC mimic, miR-214 inhibitor, or NC inhibitor, followed by the detection of cellular AMPK protein using specific AMPK antibody (Figure S2 in Supplementary Material). We found that transfection of 120 pmol of miR-214 mimic significantly reduced, while miR-214 inhibitor increased, cellular AMPK protein expression (Figure 3B), suggesting that AMPK was probably a target gene of miR-214.

**Table 2:** Six downregulated genes from transcriptomic sequencing data.

Gene	Signaling pathway	Fold change	*P* value
AMPK	AMPK signaling pathway	0.72	0.049
STAT	STAT signaling pathway	0.87	0.047
MMP9	MTOR signaling pathway	0.78	0.003
EIF4E	TNF signaling pathway	0.79	0.020
NLK	FOXO signaling pathway	0.81	0.048
STAT3	HIF-1 signaling pathway	0.86	0.047

*AMPK, adenosine 5′-monophosphate (AMP)-activated protein kinase; STAT, signal transducers and activators of transcription; MMP9, matrix metallopeptidase 9; EIF4E, eukaryotic translation initiation factor 4E; NLK, nemo-like kinase; STAT3, signal transducer and activator of transcription 3*.

Using Miranda software, two putative binding sites of miR-214 were identified at the 3′ UTR of AMPK mRNA (Figure 3C). To further confirm whether AMPK was the target gene of miR-214, we first constructed a dual-luciferase reporter plasmid pmirGLO-AMPK containing the wild-type sequence of the 3′ UTR of AMPK. Based on the plasmid pmirGLO-AMPK, we generated two mutant plasmids pmirGLO-AMPK-MUT1 and pmirGLO-AMPK-MUT2, in which the miR-214-targeted sequences were mutated (Figure 3C). These plasmids were subsequently transfected into SSN-1 cells with miR-214 mimic, NC mimic, miR-214 inhibitor, or NC inhibitor, respectively. Significant reduction in luciferase activity was observed in cells co-transfected with miR-214 mimic and the plasmid with wild-type AMPK 3′ UTR, whereas significantly increased luciferase activity was detected when transfected with miR-214 inhibitor (Figure 3D). However, the luciferase activity was not significantly altered when miR-214 mimic or inhibitor was co-transfected with the mutant plasmids harboring miR-214 seed-region mutated sequences (Figure 3D). These results indicate that AMPK is a target gene of miR-214.

### SHVV Infection Upregulates AMPK

**Figure 4:**
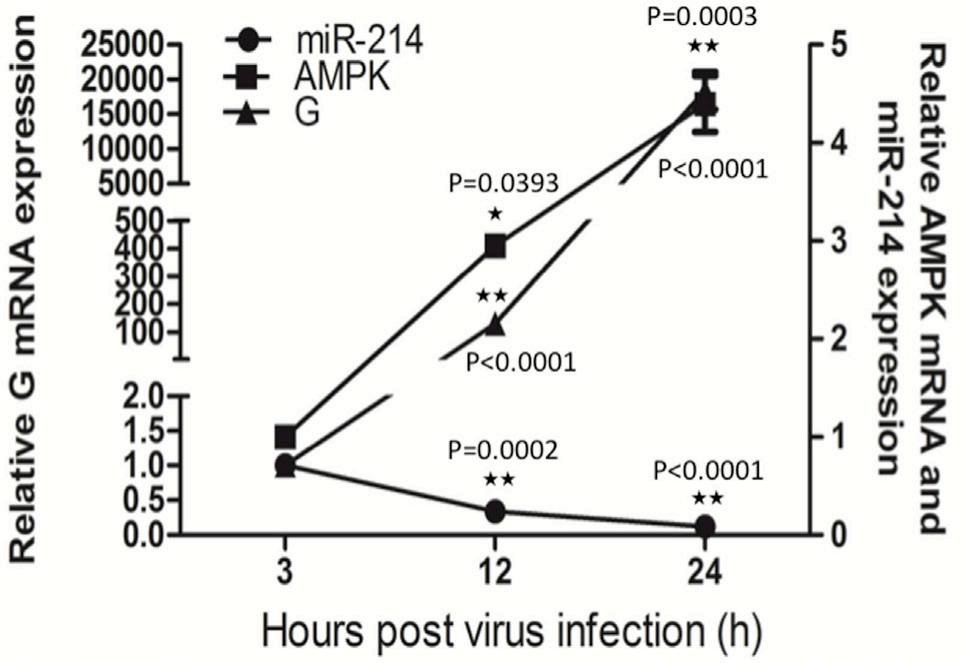
Snakehead vesiculovirus (SHVV) infection upregulates adenosine 5′-monophosphate-activated protein kinase (AMPK). Striped snakehead (SSN)-1 cells were infected with SHVV and the cells were harvested at 3, 12, and 24 h poi. The G mRNA, AMPK mRNA, and miR-214 were determined by qRT-PCR, β-actin was used as the internal control for G mRNA and AMPK mRNA, while 5S rRNA was used as the internal control for miR-214. All the data are representative of at least two independent experiments, with each determination performed in triplicate (mean ± SD). The * and **, respectively, indicate statistically significant differences (*P < 0.05; **P < 0.01).

To study the effect of SHVV infection on AMPK, SSN-1 cells were infected with SHVV and the cells were harvested at 3, 12, and 24 h poi. The G mRNA, AMPK mRNA, and miR-214 were determined by qRT-PCR. Along with the increase of G mRNA, miR-214 was steadily decreased at 12 and 24 h poi (Figure 4). This result was consistent with our previous study, in which SHVV infection downregulated miR-214 (14). In addition, AMPK mRNA was significantly increased at 12 and 24 h poi (Figure 4), suggesting that SHVV infection upregulated AMPK possibly *via* downregulating miR-214.

### Knockdown of AMPK Inhibits SHVV Replication and Promotes IFN-α Expression

**Figure 5:**
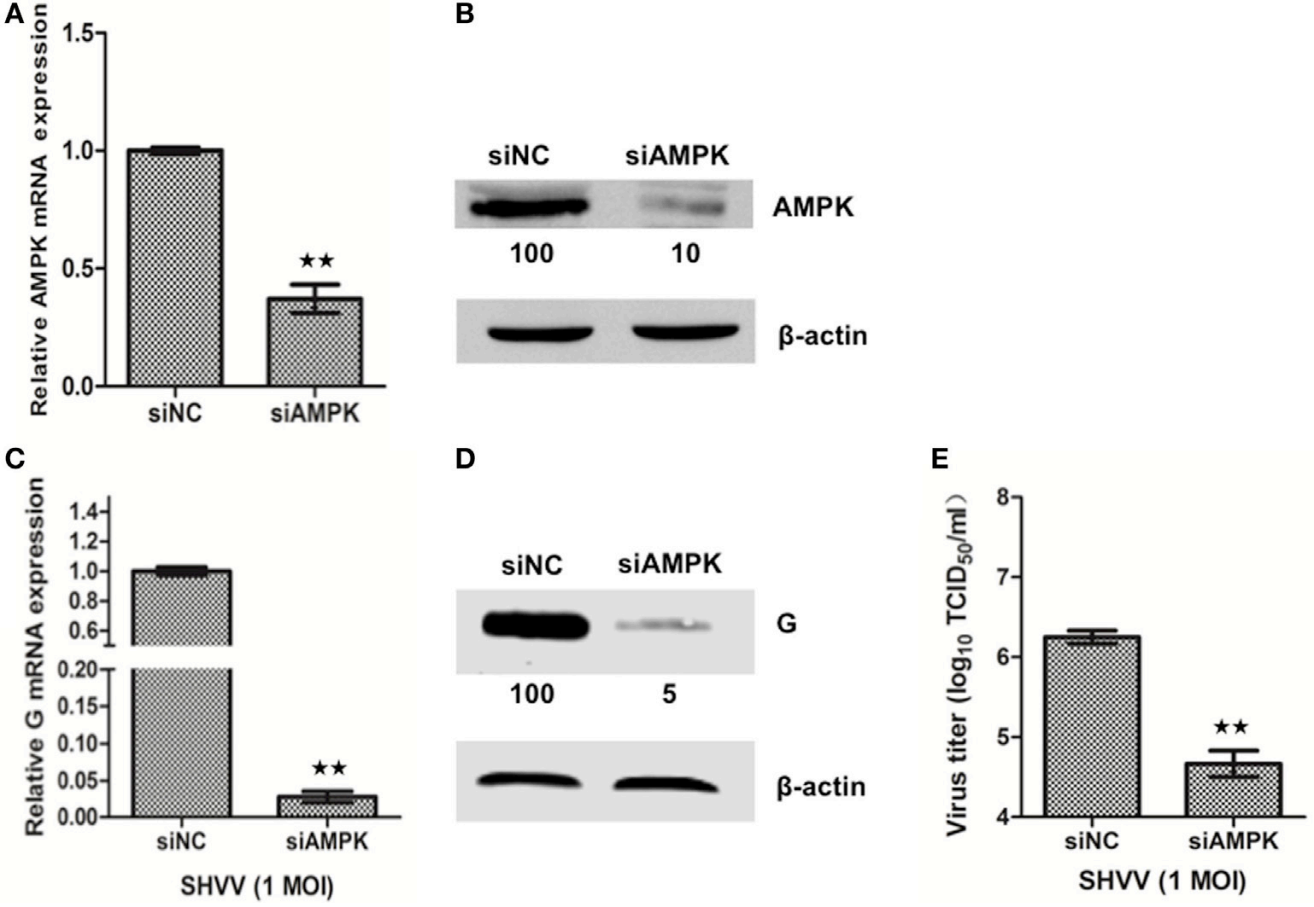
Knockdown of adenosine 5′-monophosphate-activated protein kinase (AMPK) inhibits snakehead vesiculovirus (SHVV) replication. **(A,B)** Striped snakehead (SSN)-1 cells were transfected with siNC or siAMPK, the AMPK mRNA **(A)** and protein **(B)** in SSN-1 cells was measured at 24 h post of transfection using qRT-PCR or western blot. β-actin was used as the internal control. The integrated optical densities of the protein bands were measured using Image-Pro Plus 6.0. The values of the AMPK protein bands were normalized to that of β-actin. The values of the AMPK protein band in cells transfected with siNC was set as 100. **(C–E)** SSN-1 cells were transfected with siNC or siAMPK, followed by SHVV infection. G mRNA **(C)** in SSN-1 cells was measured using qRT-PCR at 24 h poi. β-actin was used as the internal control. G Protein **(D)** was determined by western blot at 24 h poi. The integrated optical densities of the protein bands were measured using Image-Pro Plus 6.0. The values of the G protein bands were normalized to that of β-actin. The values of the G protein bands in cells transfected with siNC was set as 100. The SHVV titers **(E)** in the supernatants were measured using TCID_50_ at 24 h poi. All the data are representative of at least two independent experiments, with each determination performed in triplicate (mean ± SD). The * and **, respectively, indicate statistically significant differences (*P < 0.05; **P < 0.01).

In order to understand the role of AMPK in SHVV infection, SSN-1 cells were transfected with siAMPK or siNC, followed by SHVV infection. Transfection of siAMPK significantly reduced the mRNA and protein levels of AMPK compared to that transfected with siNC (Figures 5A,B). The effect of AMPK on SHVV replication was further evaluated by detecting viral G mRNA, G protein, and viral titer at 24 h poi. As shown in Figures 5C,D, the viral G mRNA and protein were reduced to less than 10% in siAMPK transfected cells than in siNC transfected cells. Similarly, the viral titer was decreased more than 10-fold in siAMPK group than in siNC group (Figure 5E). It can thus be suggested that knockdown of AMPK, similar to the overexpression of miR-214, inhibited SHVV replication.

In addition to affecting SHVV replication, siAMPK increased IFN-α mRNA about 10-fold (Figure S3A in Supplementary Material). To further confirm the effects of AMPK on IFN-α expression, SSN-1 cells were transfected with plasmid p3XFLAG-CMV-14 or p3XFLAG-CMV-14-AMPK, followed by SHVV infection. At 24 h poi, the cellular IFN-α mRNA was measured by qRT-PCR. The results showed that overexpression of AMPK reduced IFN-α mRNA level (Figure S3B,C in Supplementary Material). These findings suggest that AMPK negatively regulates IFN-α expression.

### Suppression of Cellular miR-214 Can Restore siAMPK-Mediated Inhibition of SHVV Replication

**Figure 6:**
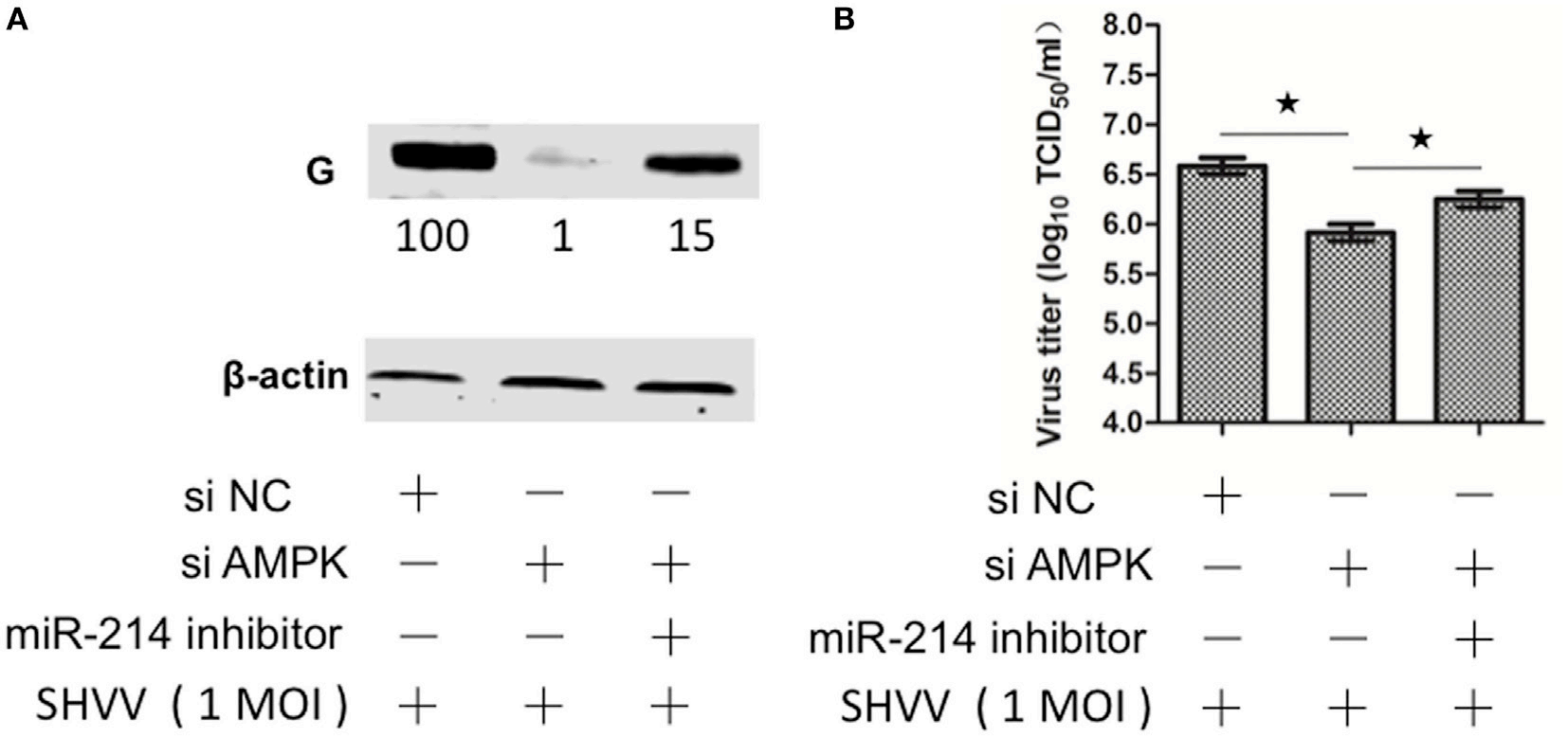
Suppression of cellular miR-214 can restore siAMPK-mediated inhibition of snakehead vesiculovirus (SHVV) replication. Striped snakehead (SSN)-1 cells were transfected with siNC, siAMPK, or siAMPK with miR-214 inhibitor, followed by SHVV infection. **(A)** The G protein was determined by western blot at 24 h poi. β-actin was used as the internal control. The integrated optical densities of the protein bands were measured using Image-Pro Plus 6.0. The values of the G protein bands were normalized to that of β-actin. The values of the G protein bands in cells transfected with siNC was set as 100. **(B)** The SHVV titers in the supernatants were measured using TCID_50_ at 24 h poi. All the data are representative of at least two independent experiments, with each determination performed in triplicate (mean ± SD). The * and **, respectively, indicate statistically significant differences (*P < 0.05; **P < 0.01).

In order to figure out whether miR-214-mediated inhibition of SHVV replication was caused by targeting AMPK, SSN-1 cells were transfected with siNC, siAMPK, or siAMPK with miR-214 inhibitor, followed by SHVV infection. The cells and supernatants were collected at 24 h poi. The viral G protein and viral titer were determined. As shown in Figure 6A, siAMPK reduced G protein level to about 1% compared to that in siNC group. However, addition of miR-214 inhibitor restored the G protein level to 15%. Similarly, the viral titer was significantly decreased by siAMPK, which was partially restored by the transfection with miR-214 inhibitor (Figure 6B). Overall, these findings indicate that miR-214 inhibits SHVV replication at least partially due to its targeting AMPK.

## Discussion

Host miRNA has emerged as both responsive factor and modulator of virus infection. In detail, host miRNA expression is commonly altered in response to virus infection, and the altered miRNA in turn modulate virus infection (1, 6, 9–11, 22). Santhakumar et al. have revealed that miR-214 was downregulated upon several human and mammalian viruses infection, and in turn, miR-214 inhibited the replication of these viruses, suggesting that miR-214 acted as a broad antiviral miRNA (22). Our previous study has revealed that miR-214 was downregulated upon a fish rhabdovirus SHVV infection (14), and overexpression of miR-214 inhibited SHVV replication *via* targeting N and P genes of SHVV (15). As the genomes of these miR-214-inhibited viruses share little sequence similarity, it is speculated that miR-214 might target host factors that are required by multiple viruses. Thereby, the aim of this study is to identify miR-214-targeted host factors and the related antiviral mechanism.

The mechanisms of miRNA-mediated regulation of virus replication have attracted much attention these years. Growing evidences have demonstrated that miRNAs could inhibit virus replication by targeting host factors that were critically important for virus replication (23–32). For example, host eukaryotic translation elongation factor 1A1 (EEF1A1) can interact with NS3 and NS5 proteins of Japanese encephalitis (JEV) to form a complex that is essential for JEV replication, miR-33a can target EEF1A1 and reduce its expression, thus suppressing JEV replication (31). In addition, miRNAs can also target host factors that positively or negatively regulate type I interferon expression or the following signaling (6, 9, 33–44). In the current study, we found that miR-214, the important cancer development regulator (45, 46), inhibited SHVV replication by regulating host IFN-α expression (15) (Figure 2). Many miRNAs have been identified as type I interferon regulators, including miR-373 (6), miR-466l (34), miR-155 (36), miR-15b (38), miR-526a (39), miR-223 (41), and miR-146a (42–44). Here, miR-214 was identified as a novel type I interferon regulator.

Adenosine 5′-monophosphate-activated protein kinase has been extensively studied as a pivotal regulator of cellular energy metabolism (47). Recent studies have revealed that AMPK was involved in the regulation of virus replication (18). Inhibition of AMPK severely attenuated HCMV replication, suggesting that AMPK was required for HCMV replication (48, 49). In the current study, knockdown of AMPK inhibited SHVV replication, suggesting that AMPK was beneficial for SHVV replication (Figure 5). However, activation of AMPK has been reported to restrict hepatitis B virus (HBV) production, suggesting that AMPK was disadvantageous for HBV replication (50). Therefore, AMPK played different roles in different viruses infection. Although our study demonstrated that miR-214-mediated inhibition of SHVV replication was at least partially due to targeting it’s target gene AMPK, the broad antiviral property of miR-214 was probably not caused by its targeting AMPK because AMPK not only promoted but also restricted some viruses replication (50). In the current study, in addition to AMPK, five other host factors, including STAT, MMP9, EIF4E, NLK, and STAT3, have also been identified as potential target genes of miR-214 (Figure 3A). Therefore, identifying the host target gene of miR-214 that was responsible for the broad antiviral property of miR-214 needed to be investigated further.

Recently, AMPK has been indicated to regulate type I interferon expression. Inhibition of AMPK was observed to suppress IFN-β induction (51). In the current study, knockdown of AMPK promoted, whereas overexpression of AMPK inhibited, IFN-α expression (Figure S3 in Supplementary Material). Moreover, our study revealed that knockdown of AMPK promoted poly (I:C)-induced IFN-α expression (Figure S4 in Supplementary Material). These findings suggested that AMPK could regulate type I interferon expression. Our previous study has indicated that viral P protein of SHVV inhibited IFN-α expression, and miR-214 could target the P gene and thus suppressed P-mediated inhibition of IFN-α expression (15). As AMPK was also identified as a target gene of miR-214, It’s speculated that miR-214-mediated regulation of IFN-α expression might also be due to targeting AMPK. Taken together, our studies suggested that miR-214 promoted IFN-α expression by targeting not only viral P gene but also host AMPK gene. Despite these promising results, further studies are needed to investigate how P and AMPK regulated host IFN-α expression.

## Author Contributions

JT and LL designed the research. CZ, SF, WZ, NC, AH, WC, and LZ performed the experiments, contributed to the data collection and statistical analysis. JT, LL, XL, and JL finalized the paper writing.

## Conflict of Interest Statement

The authors declare that the research was conducted in the absence of any commercial or financial relationships that could be construed as a potential conflict of interest.

## References

[1] Deng W Zhang X Ma Z Lin Y Lu M. MicroRNA-125b-5p mediates post-transcriptional regulation of hepatitis B virus replication via the LIN28B/let-7 axis. RNA Biol (2017) 14(10):1389–98. 10.1080/15476286.2017.1293770 28267418 PMC5711457

[2] Riess M Fuchs NV Idica A Hamdorf M Flory E Pedersen IM Interferons induce expression of SAMHD1 in monocytes through down-regulation of miR-181a and miR-30a. J Biol Chem (2017) 292(1):264–77. 10.1074/jbc.M116.752584 27909056 PMC5217685

[3] Smith JL Jeng S McWeeney SK Hirsch AJ. A microRNA screen identifies the Wnt signaling pathway as a regulator of the interferon response during flavivirus infection. J Virol (2017) 91(8):e02388–16. 10.1128/JVI.02388-16 28148804 PMC5375670

[4] Tundup S Kandasamy M Perez JT Mena N Steel J Nagy T Endothelial cell tropism is a determinant of H5N1 pathogenesis in mammalian species. PLoS Pathog (2017) 13(3):e1006270. 10.1371/journal.ppat.1006270 28282445 PMC5362246

[5] Yang Y Liu Y Xue J Yang Z Shi Y Shi Y MicroRNA-141 targets Sirt1 and inhibits autophagy to reduce HBV replication. Cell Physiol Biochem (2017) 41(1):310–22. 10.1159/000456162 28135713

[6] Chen J Shi X Zhang X Wang A Wang L Yang Y MicroRNA 373 facilitates the replication of porcine reproductive and respiratory syndrome virus by its negative regulation of type I interferon induction. J Virol (2017) 91(3):e1311–6. 10.1128/JVI.01311-16 27881653 PMC5244336

[7] Hazra B Kumawat KL Basu A. The host microRNA miR-301a blocks the IRF1-mediated neuronal innate immune response to Japanese encephalitis virus infection. Sci Signal (2017) 10(466):eaaf5185. 10.1126/scisignal.aaf5185 28196914

[8] Zuo H Yuan J Chen Y Li S Su Z Wei E A microRNA-mediated positive feedback regulatory loop of the NF-kappaB pathway in *Litopenaeus vannamei*. J Immunol (2016) 196(9):3842–53. 10.4049/jimmunol.1502358 26994223

[9] Zhang Q Huang C Yang Q Gao L Liu HC Tang J MicroRNA-30c modulates type I IFN responses to facilitate porcine reproductive and respiratory syndrome virus infection by targeting JAK1. J Immunol (2016) 196(5):2272–82. 10.4049/jimmunol.1502006 26826240

[10] Zheng Q Hou J Zhou Y Yang Y Cao X. Type I IFN-inducible downregulation of microRNA-27a feedback inhibits antiviral innate response by upregulating Siglec1/TRIM27. J Immunol (2016) 196(3):1317–26. 10.4049/jimmunol.1502134 26700765

[11] Kanokudom S Vilaivan T Wikan N Thepparit C Smith DR Assavalapsakul W. miR-21 promotes dengue virus serotype 2 replication in HepG2 cells. Antiviral Res (2017) 142:169–77. 10.1016/j.antiviral.2017.03.020 28365456

[12] Liu XD Wen Y Hu XQ Wang WW Liang XF Li J Breaking the host range: mandarin fish is susceptible to a vesiculovirus derived from snakehead fish. J Gen Virol (2015) 96:775–81. 10.1099/vir.0.000037 25537376

[13] Dietzgen RG Kondo H Goodin MM Kurath G Vasilakis N. The family Rhabdoviridae: mono- and bipartite negative-sense RNA viruses with diverse genome organization and common evolutionary origins. Virus Res (2017) 227:158–70. 10.1016/j.virusres.2016.10.010 27773769 PMC5124403

[14] Liu XD Tu JG Yuan JF Liu XQ Zhao LJ Dawar FU Identification and characterization of microRNAs in snakehead fish cell line upon snakehead fish vesiculovirus infection. Int J Mol Sci (2016) 17(2):E154. 10.3390/ijms17020154 26821019 PMC4783888

[15] Zhang C Yi L Feng S Liu X Su J Lin L MicroRNA miR-214 inhibits snakehead vesiculovirus replication by targeting the coding regions of viral N and P. J Gen Virol (2017) 98(7):1611–9. 10.1099/jgv.0.000854 28699870

[16] Chu Q Sun Y Cui J Xu T Inducible microRNA-214 contributes to the suppression of NF-kappaB-mediated inflammatory response via targeting myd88 gene in fish. J Biol Chem (2017) 292(13):5282–90. 10.1074/jbc.M117.777078 28235799 PMC5392675

[17] Xiao B Sanders MJ Underwood E Heath R Mayer FV Carmena D Structure of mammalian AMPK and its regulation by ADP. Nature (2011) 472(7342):230–3. 10.1038/nature09932 21399626 PMC3078618

[18] Mesquita I Moreira D Sampaio-Marques B Laforge M Cordeiro-da-Silva A Ludovico P AMPK in pathogens. EXS (2016) 107:287–323. 10.1007/978-3-319-43589-3 27812985

[19] Moreira D Silvestre R Cordeiro-da-Silva A Estaquier J Foretz M Viollet B. AMP-activated protein kinase as a target for pathogens: friends or foes? Curr Drug Targets (2016) 17(8):942–53. 10.2174/1389450116666150416120559 25882224 PMC5387108

[20] Ji WT Lee LH Lin FL Wang L Liu HJ. AMP-activated protein kinase facilitates avian reovirus to induce mitogen-activated protein kinase (MAPK) p38 and MAPK kinase 3/6 signalling that is beneficial for virus replication. J Gen Virol (2009) 90(Pt 12):3002–9. 10.1099/vir.0.013953-0 19656961

[21] Moser TS Schieffer D Cherry S. AMP-activated kinase restricts rift valley fever virus infection by inhibiting fatty acid synthesis. PLoS Pathog (2012) 8(4):e1002661. 10.1371/journal.ppat.1002661 22532801 PMC3330235

[22] Santhakumar D Forster T Laqtom NN Fragkoudis R Dickinson P Abreu-Goodger C Combined agonist-antagonist genome-wide functional screening identifies broadly active antiviral microRNAs. Proc Natl Acad Sci U S A (2010) 107(31):13830–5. 10.1073/pnas.1008861107 20643939 PMC2922253

[23] Huang JY Chou SF Lee JW Chen HL Chen CM Tao MH MicroRNA-130a can inhibit hepatitis B virus replication via targeting PGC1 alpha and PPAR gamma. RNA (2015) 21(3):385–400. 10.1261/rna.048744.114 25595716 PMC4338335

[24] Slonchak A Hussain M Torres S Asgari S Khromykh AA. Expression of mosquito microRNA Aae-miR-2940-5p is downregulated in response to West Nile virus infection to restrict viral replication. J Virol (2014) 88(15):8457–67. 10.1128/JVI.00317-14 24829359 PMC4135961

[25] Ouda R Onomoto K Takahasi K Edwards MR Kato H Yoneyama M Retinoic acid-inducible gene I-inducible miR-23b inhibits infections by minor group rhinoviruses through down-regulation of the very low density lipoprotein receptor. J Biol Chem (2011) 286(29):26210–9. 10.1074/jbc.M111.229856 21642441 PMC3138319

[26] Gao L Guo XK Wang L Zhang Q Li N Chen XX MicroRNA 181 suppresses porcine reproductive and respiratory syndrome virus (PRRSV) infection by targeting PRRSV receptor CD163. J Virol (2013) 87(15):8808–12. 10.1128/JVI.00718-13 23740977 PMC3719804

[27] Cheng M Si Y Niu Y Liu X Li X Zhao J High-throughput profiling of alpha interferon- and interleukin-28B-regulated microRNAs and identification of let-7s with anti-hepatitis C virus activity by targeting IGF2BP1. J Virol (2013) 87(17):9707–18. 10.1128/JVI.00802-13 23824794 PMC3754137

[28] Zheng SQ Li YX Zhang Y Li X Tang H. MiR-101 regulates HSV-1 replication by targeting ATP5B. Antiviral Res (2011) 89(3):219–26. 10.1016/j.antiviral.2011.01.008 21291913

[29] Fu YR Liu XJ Li XJ Shen ZZ Yang B Wu CC MicroRNA miR-21 attenuates human cytomegalovirus replication in neural cells by targeting Cdc25a. J Virol (2015) 89(2):1070–82. 10.1128/JVI.01740-14 25378484 PMC4300626

[30] Loveday EK Diederich S Pasick J Jean F. Human microRNA-24 modulates highly pathogenic avian-origin H5N1 influenza A virus infection in A549 cells by targeting secretory pathway furin. J Gen Virol (2015) 96(Pt 1):30–9. 10.1099/vir.0.068585-0 25234642

[31] Chen Z Ye J Ashraf U Li Y Wei S Wan S MicroRNA-33a-5p modulates japanese encephalitis virus replication by targeting eukaryotic translation elongation factor 1A1. J Virol (2016) 90(7):3722–34. 10.1128/JVI.03242-15 26819305 PMC4794666

[32] Hu Y Jiang L Lai W Qin Y Zhang T Wang S MicroRNA-33a disturbs influenza A virus replication by targeting ARCN1 and inhibiting viral ribonucleoprotein activity. J Gen Virol (2016) 97(1):27–38. 10.1099/jgv.0.000311 26498766

[33] Buggele WA Horvath CM. MicroRNA profiling of sendai virus-infected A549 cells identifies miR-203 as an interferon-inducible regulator of IFIT1/ISG56. J Virol (2013) 87(16):9260–70. 10.1128/JVI.01064-13 23785202 PMC3754065

[34] Li Y Fan X He X Sun H Zou Z Yuan H MicroRNA-466l inhibits antiviral innate immune response by targeting interferon-alpha. Cell Mol Immunol (2012) 9(6):497–502. 10.1038/cmi.2012.35 23042536 PMC4002216

[35] Bhanja Chowdhury J Shrivastava S Steele R Di Bisceglie AM Ray R Ray RB. Hepatitis C virus infection modulates expression of interferon stimulatory gene IFITM1 by upregulating miR-130A. J Virol (2012) 86(18):10221–5. 10.1128/JVI.00882-12 22787204 PMC3446586

[36] Wang P Hou J Lin L Wang C Liu X Li D Inducible microRNA-155 feedback promotes type I IFN signaling in antiviral innate immunity by targeting suppressor of cytokine signaling 1. J Immunol (2010) 185(10):6226–33. 10.4049/jimmunol.1000491 20937844

[37] Li Y Xie J Xu X Wang J Ao F Wan Y MicroRNA-548 down-regulates host antiviral response via direct targeting of IFN-lambda1. Protein Cell (2013) 4(2):130–41. 10.1007/s13238-012-2081-y 23150165 PMC4875363

[38] Zhu B Ye J Nie Y Ashraf U Zohaib A Duan X MicroRNA-15b modulates Japanese encephalitis virus-mediated inflammation via targeting RNF125. J Immunol (2015) 195(5):2251–62. 10.4049/jimmunol.1500370 26202983

[39] Xu C He X Zheng Z Zhang Z Wei C Guan K Downregulation of microRNA miR-526a by enterovirus inhibits RIG-I-dependent innate immune response. J Virol (2014) 88(19):11356–68. 10.1128/JVI.01400-14 25056901 PMC4178780

[40] Gao D Zhai A Qian J Li A Li Y Song W Down-regulation of suppressor of cytokine signaling 3 by miR-122 enhances interferon-mediated suppression of hepatitis B virus. Antiviral Res (2015) 118:20–8. 10.1016/j.antiviral.2015.03.001 25766860

[41] Chen L Song Y He L Wan X Lai L Dai F MicroRNA-223 promotes type I interferon production in antiviral innate immunity by targeting Forkhead box protein O3 (FOXO3). J Biol Chem (2016) 291(28):14706–16. 10.1074/jbc.M115.700252 27226534 PMC4938189

[42] Wu S He L Li Y Wang T Feng L Jiang L miR-146a facilitates replication of dengue virus by dampening interferon induction by targeting TRAF6. J Infect (2013) 67(4):329–41. 10.1016/j.jinf.2013.05.003 23685241

[43] Ho BC Yu IS Lu LF Rudensky A Chen HY Tsai CW Inhibition of miR-146a prevents enterovirus-induced death by restoring the production of type I interferon. Nat Commun (2014) 5:3344. 10.1038/ncomms4344 24561744

[44] Hou J Wang P Lin L Liu X Ma F An H MicroRNA-146a feedback inhibits RIG-I-dependent Type I IFN production in macrophages by targeting TRAF6, IRAK1, and IRAK2. J Immunol (2009) 183(3):2150–8. 10.4049/jimmunol.0900707 19596990

[45] Liu Y Zhou H Ma L Hou Y Pan J Sun C MiR-214 suppressed ovarian cancer and negatively regulated semaphorin 4D. Tumour Biol (2016) 37(6):8239–48. 10.1007/s13277-015-4708-0 26718213

[46] Sharma T Hamilton R Mandal CC miR-214: a potential biomarker and therapeutic for different cancers. Future Oncol (2015) 11(2):349–63. 10.2217/fon.14.193 25591843

[47] Chen M Liu J Yang L Ling W AMP-activated protein kinase regulates lipid metabolism and the fibrotic phenotype of hepatic stellate cells through inhibition of autophagy. FEBS Open Bio (2017) 7(6):811–20. 10.1002/2211-5463.12221 PMC5458462 28593136

[48] McArdle J Moorman NJ Munger J. HCMV targets the metabolic stress response through activation of AMPK whose activity is important for viral replication. PLoS Pathog (2012) 8(1):e1002502. 10.1371/journal.ppat.1002502 22291597 PMC3266935

[49] Terry LJ Vastag L Rabinowitz JD Shenk T Human kinome profiling identifies a requirement for AMP-activated protein kinase during human cytomegalovirus infection. Proc Natl Acad Sci U S A (2012) 109(8):3071–6. 10.1073/pnas.1200494109 22315427 PMC3286917

[50] Xie N Yuan K Zhou L Wang K Chen HN Lei Y PRKAA/AMPK restricts HBV replication through promotion of autophagic degradation. Autophagy (2016) 12(9):1507–20. 10.1080/15548627.2016.1191857 27305174 PMC5082782

[51] Prantner D Perkins DJ Vogel SN. AMP-activated kinase (AMPK) promotes innate immunity and antiviral defense through modulation of stimulator of interferon genes (STING) signaling. J Biol Chem (2017) 292(1):292–304. 10.1074/jbc.M116.763268 27879319 PMC5217687

