# Supplementary material for: MicroRNA miR-214 Inhibits Snakehead Vesiculovirus Replication by Promoting IFN-α Expression via Targeting Host Adenosine 5′-Monophosphate-Activated Protein Kinase
Source: Front Immunol. 2017 Dec 11;8:1775. doi: 10.3389/fimmu.2017.01775 (PMC5732478; doi:10.3389/fimmu.2017.01775)
Supplement: Supplementary file 1 [file Presentation_1.PDF]

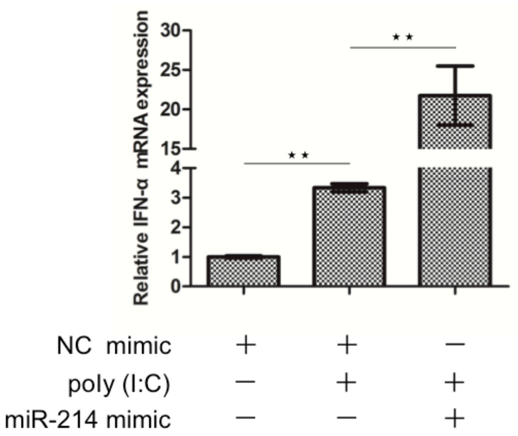

Figure S1. The effect of miR-214 on poly (I:C)-induced IFN-α expression. The SSN-1 cells were transfected with NC mimic, poly (I:C) with NC mimic, or poly (I:C) with miR-214 mimic for 24 h. IFN-α mRNA levels in cells were determined by qRT-PCR, β-actin was used as the internal control. All the data are representative of at least two independent experiments, with each determination performed in triplicate (mean ± SD). The * and ** respectively indicate statistically significant differences (*, $p < 0.05$; **, $p < 0.01$).

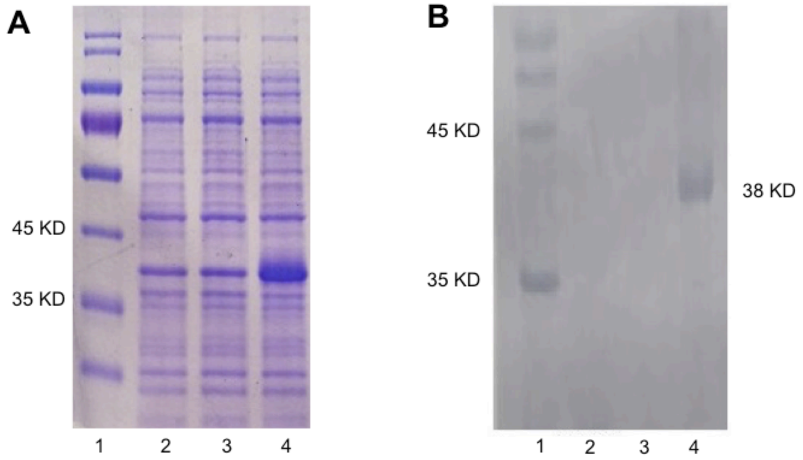

Figure S2. The specificity of AMPK antibody. (A) The prokaryotic expression of AMPK. Line 1: marker; line 2: pET-32a; line 3: pET-32a-AMPK before IPTG induction; line 4: pET-32a-AMPK after IPTG induction. (B) Western blot shows the specificity of AMPK antibody. Line 1: marker; line 2: pET-32a; line 3: pET-32a-AMPK before IPTG induction; line 4: pET-32a-AMPK after IPTG induction with 10,000 dilution.

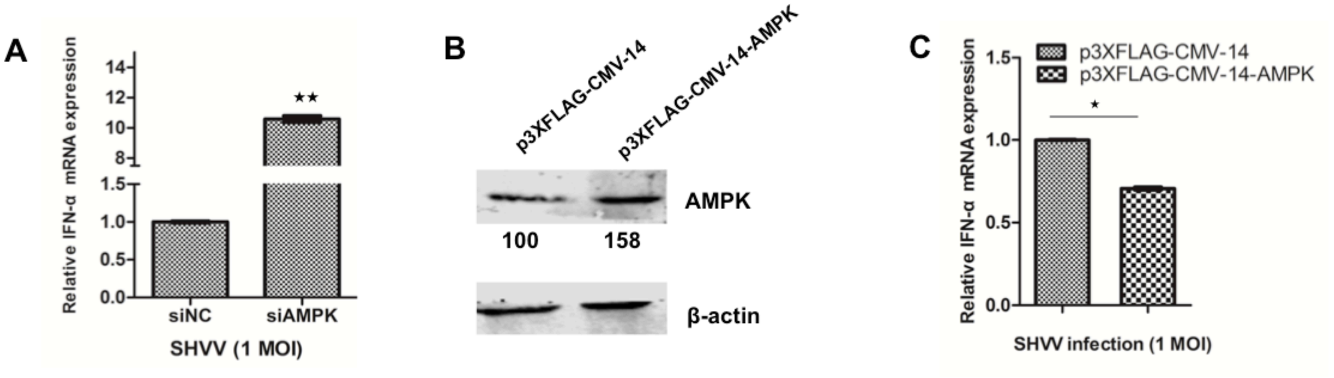

Figure S3. The effect of AMPK on IFN-α expression. (A) SSN-1 cells were transfected with siNC or siAMPK, followed by SHVV infection. The IFN-α mRNA in SSN-1 cells was measured using qRT-PCR at 24 h poi. β-actin was used as the internal control. (B) SSN-1 cells were transfected with p3XFLAG-CMV-14 or p3XFLAG-CMV-14-AMPK, the cells were collected at 24 h post of transfection and AMPK protein was detected by western blot. The integrated optical densities of the protein bands were measured using Image-Pro Plus 6.0. The values of the AMPK protein bands were normalized to that of β-actin. The values of the AMPK protein band in cells transfected with p3XFLAG-CMV-14 was set as 100. (C) SSN-1 cells were transfected with p3XFLAG-CMV-14 or p3XFLAG-CMV-14-AMPK, followed by SHVV infection. At 24 h poi, the cellular IFN-α mRNA was measured using qRT-PCR. β-actin was used as the internal control. All the data are representative of at least two independent experiments, with each determination performed in triplicate (mean ± SD). The * and ** respectively indicate statistically significant differences (*, $p < 0.05$; **, $p < 0.01$).

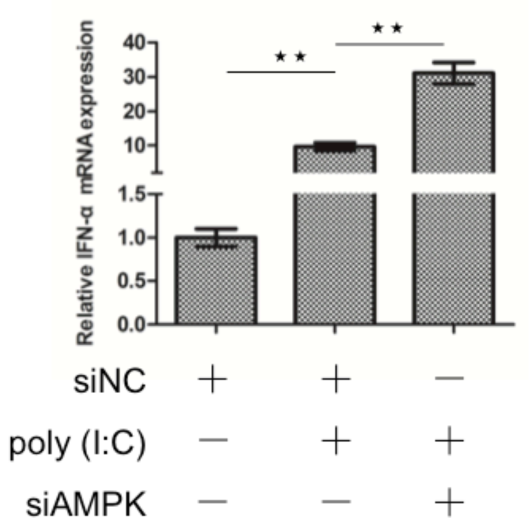

Figure S4. Knockdown of AMPK promotes IFN-α production induced by poly (I:C). (A) SSN-1 cells were transfected with siNC, siNC and poly (I:C), or siAMPK and poly (I:C). At 24 h post of transfection, the IFN-α mRNA was detected by qRT-PCR. β-actin was used as the internal control. All the data are representative of at least two independent experiments, with each determination performed in triplicate (mean ± SD). The * and ** respectively indicate statistically significant differences (*, $p < 0.05$; **, $p < 0.01$).
